# A multi-center, cross-sectional questionnaire survey in Japan (KOBE study) exploring factors associated with primary focal hyperhidrosis

**DOI:** 10.3389/fmed.2026.1739715

**Published:** 2026-02-09

**Authors:** Takeshi Fukumoto, Marie Ohata, Yukari Matsumoto, Masashi Ashida, Tetsuya Ikeda, Yusuke Inoue, Satoshi Ogawa, Shuntaro Oniki, Tsuneyoshi Kamo, Takeshi Kozaru, Daisuke Sakai, Masanobu Sakagachi, Yoshihito Sasaki, Hirofumi Sato, Haruki Jimbo, Shingo Tamura, Tsuyoshi Numata, Kazuhito Hayashibe, Susumu Harada, Toshinori Bito, Ayako Fukumoto, Tatsuya Horikawa, Chihiro Honda, Junji Yamashita, Atsushi Yamamoto, Hiromichi Okatsu, Takashi Hashimoto, Hiroshi Miyama, Akiharu Kubo

**Affiliations:** 1 Division of Dermatology, Department of Internal Related, Kobe University Graduate School of Medicine, Kobe, Japan; 2 Department of Dermatology, Graduate School of Medical Science, Kyoto Prefectural University of Medicine, Kyoto, Japan; 3 Ashida Skin Clinic, Sasayama, Japan; 4 Ikeda Skin Clinic, Kobe, Japan; 5 Inoue Dermatology Clinic, Himeji, Japan; 6 Ogawa Skin Clinic, Kakogawa, Japan; 7 Oniki Dermatological Clinic, Akashi, Japan; 8 Kamo Skin Clinic, Kobe, Japan; 9 Kozaru Skin Clinic, Kato, Japan; 10 Sakai Dermatology Clinic, Miki, Japan; 11 Sakaguchi Skin Clinic, Ibaraki, Japan; 12 Nishiakashi Sasaki Dermatology, Akashi, Japan; 13 Sato Skin Clinic, Tamba, Japan; 14 Jimbo Dermatology Clinic, Kobe, Japan; 15 Tamura Skin Clinic, Kobe, Japan; 16 Numata Skin Clinic, Kobe, Japan; 17 Hayashibe Dermatology Clinic, Sanda, Japan; 18 Harada Skin Clinic, Nishinomiya, Japan; 19 Bito Dermatology Clinic, Kobe, Japan; 20 Honda Dermatological Clinic, Kobe, Japan; 21 Ueda Hifuka Clinic, Kakogun, Japan; 22 Pure Skin Clinic, Kobe, Japan; 23 Yamamoto Hifuka Kampo Clinic, Himeji, Japan; 24 Kaken Pharmaceutical Co., Ltd., Tokyo, Japan

**Keywords:** associated factor, hospital anxiety and depression scale score, KOBE study, primary focal hyperhidrosis, questionnaire survey

## Abstract

**Background/aim:**

Primary focal hyperhidrosis (PFH) is defined as a condition characterized by excessive sweating in localized areas, which causes patients to experience difficulties in daily life, regardless of temperature or psychological stress. Previous surveys in Japan have revealed that the majority of patients with PFH may not visit medical institutions. Identifying the factors potentially associated with PFH is useful for detecting unmedicated patients and providing appropriate medical interventions. In this study, we explored factors associated with PFH in a multi-center, cross-sectional questionnaire survey (KOBE study).

**Methods:**

This study enrolled patients aged 5–64 years who visited 1 of the 24 dermatological institutions in Japan between April and July 2024 and completed a questionnaire (registered at the Japan Registry of Clinical Trials: jRCT1050250083). A combination of univariate and multivariate logistic regression analyses was performed to explore the associated factors.

**Results:**

A total of 3,617 participants were included in the analysis. The prevalence of PFH was 15.0% (544 of 3,617 participants). Among the potential associated factors, the odds ratios (ORs) were higher in order of axillary osmidrosis (OR = 5.440), psoriasis (OR = 1.830), wet earwax (OR = 1.780), a definite Hospital Anxiety and Depression Scale-Anxiety (HADS-A) score (OR = 1.780), a doubtful HADS-A score (OR = 1.460), and smoking (OR = 1.450). Receiver operating characteristic (ROC) curve analysis indicated that a HADS-A score of 6 was the optimal cutoff value for suspecting PFH.

**Conclusion:**

These findings may aid in detecting unmedicated potential patients in routine clinical practice and promoting active intervention for the disease, ultimately improving the quality of life and well-being of patients with PFH.

## 1 Introduction

Primary focal hyperhidrosis (PFH) is defined as a condition characterized by excessive sweating in localized areas, such as the head/face, palmar, soles, and axillary regions, which causes patients to experience difficulties in daily life, regardless of temperature or psychological stress (1). In 2020, a web-based epidemiological questionnaire survey was conducted in Japan. The survey reported that the prevalence of PFH was 10.0% among 60,969 individuals, with the highest prevalence observed in the axillary region (5.9%), followed by the head/face (3.6%), palms (2.9%), and soles (2.3%) (2). PFH has been reported to severely affect patients’ daily lives, including emotional well-being, interpersonal relationships, leisure activities, personal hygiene, work productivity, and self-esteem (3). Patients with PFH commonly suffer from sweaty stains on clothing, shoes, or touched objects; visible sweat drops on the forehead; wet handshakes; or sensation of body odor (4); therefore, PFH is not merely a physiological phenomenon, but rather a dermatological condition accompanied by visually recognizable symptoms that impose a significant impact on patients’ social lives (visible stigma) (5). A cost-of-illness survey for axillary hyperhidrosis in Japan revealed that overall work impairment among working patients is 30.52%, and the activity impairment among full-time housewives is 49.05%. The survey also estimated that the societal loss associated with the productivity loss is JPY¥312 billion per month (6).

Despite the substantial burden, the majority of patients with PFH remain untreated. According to the aforementioned epidemiological survey, only 4.6% of individuals with PFH have consulted a physician (2). Another web-based questionnaire survey in Japan reported that only 9.5% of respondents with severe axillary hyperhidrosis visit a medical facility (7). Outside Japan, reports from Germany and the United States have indicated that the consultation rates for primary hyperhidrosis are 27 and 51%, respectively (8, 9). These findings suggest that the consultation rate in Japan is lower than that in the United States and Europe. Currently, the majority of patients with PFH do not seek medical care and, consequently, do not receive appropriate treatment from healthcare professionals, especially in Japan. It is therefore important to identify unmedicated potential patients with PFH and provide them with appropriate medical care to improve their quality of life and well-being. Recognizing the potential factors associated with PFH is useful for detecting unmedicated patients and providing these patients with appropriate medical interventions. Patients with hyperhidrosis have a significantly higher prevalence of anxiety disorders and depression than those without the condition (10). Recent epidemiological studies have also shown that depression is a common comorbidity in hyperhidrosis (11). Another report has indicated a familial history of hyperhidrosis, suggesting that some genetic factors may play a role in certain patients (12). However, the cause of PFH remains unknown, and evidence of its potential associated factors is limited. In this study, we aimed to explore the factors associated with PFH in a multi-center, cross-sectional questionnaire survey in Japan (KOBE study).

## 2 Methods

### 2.1 Study design

This study was a multi-center, cross-sectional questionnaire survey of 3,617 participants included in the analysis (KOBE study). The objectives were to explore factors associated with PFH and to determine the prevalence of the disease stratified by participant background characteristics. A survey was conducted at 24 dermatological institutions in Japan. The study has been registered with the Japan Registry of Clinical Trials (jRCT1050250083). This study was approved by the Institutional Review Board of Kobe University Graduate School of Medicine (approval number: B230239) and was conducted in accordance with the principles of the Declaration of Helsinki and the ethical guidelines for medical and health research involving human subjects. Written informed consent was obtained from all participants in this study. For patients under the age of 18 years, written consent was also obtained from their parents or guardians in addition to the patients themselves, and the parent or guardian was permitted to respond to the questionnaire survey on behalf of the patient.

### 2.2 Study participants

All patients aged 5–64 years who visited 1 of the 24 dermatological institutions in Japan between April and July 2024, who agreed to participate in the study and who completed the questionnaire, were included in this study. Participants were excluded if they had any of the following conditions or treatments: (i) dementia requiring treatment, (ii) psychological disorder, (iii) coma, (iv) use of immunosuppressants or corticosteroids, (v) anti-cancer treatment for advanced cancer, and (vi) viral infection requiring treatment.

### 2.3 Survey items of questionnaire

To determine the presence of PFH symptoms, the survey included the following questions:

(i) “Have you been experiencing excessive sweating in any of the following areas—head/face, palmar, soles or axillary for more than 6 months?”; (ii) “Please select any of the following conditions that explain your excessive sweating—bilateral and relatively symmetric, impairs daily activities, frequency of at least one episode per week, age of onset less than 25 years, positive family history, or cessation of focal sweating during sleep”; and (iii) “Please select any of the following diseases for which you are receiving a therapy—obesity, menopausal disorders, Basedow’s disease, pheochromocytoma, thyroid dysfunction, cerebral infarction, acromegaly, Parkinson’s disease, hypoglycemia, or carcinoid tumors.” According to the disease definition provided in the Japanese clinical guideline for primary focal hyperhidrosis (1, 13), participants were considered to have PFH symptoms if they (1) answered “Yes” to question (i), (2) selected two or more conditions in question (2), and (3) did not select any of the diseases listed in question (3), thereby excluding secondary focal hyperhidrosis.

To explore factors associated with PFH, the survey collected comprehensive information, including participant background factors (age and sex), Hospital Anxiety and Depression Scale-Anxiety (HADS-A) and HADS-Depression (HADS-D) scores (14, 15), and lifestyle and daily habits, including smoking, alcohol consumption, adequate sleep, regular exercise, wet earwax, skipping breakfast ≥3 days a week, daily vegetable intake, preference for Japanese-style meals, daily use of visual display terminals [VDTs], customer service occupation, jobs requiring uniforms, and daily use of public transportation. The survey also inquired about subjective symptoms, including headache, dizziness, low-grade fever, palpitations, decreased appetite, constipation/diarrhea, nausea, tinnitus, irritability/anxiety, back and lower back pain, neck and shoulder pain/stiffness, numbness in the hands and feet, irregular menstruation/erectile dysfunction, and shortness of breath. In addition, participants were asked about dermatological conditions, such as atopic dermatitis, acne, axillary osmidrosis, asteatosis (dry skin), psoriasis, dandruff, tinea infections (body, foot, and nail), rosacea, seborrheic dermatitis, skin cancer, and hand eczema, and non-dermatological conditions, such as cancer, dyslipidemia, migraine, hypertension, gastroesophageal reflux disease, anxiety disorder, depression, type 2 diabetes mellitus, bronchial asthma, and sleep apnea syndrome. The survey items on lifestyle, daily habits, and subjective symptoms were selected with reference to items used in Japanese health checkups (16, 17), including those deemed inappropriate for this survey. The survey items regarding skin diseases and non-skin diseases were adopted, referring to a previous epidemiological survey on PFH in Japan (18). Participants were asked to indicate the presence or absence of each condition or whether their condition corresponded to the content of each question.

### 2.4 Outcomes

The primary outcome of this study was to explore the factors associated with PFH. The secondary outcomes were to determine the prevalence of the disease stratified by the participants’ background characteristics. Additionally, if HADS scores were identified as significant associated factors consistent with the primary outcome, we estimated their optimal cutoff values for suspecting PFH, based on previous reports suggesting that anxiety disorder and depression may be related to PFH (10, 11).

### 2.5 Statistical analysis

The prevalence of PFH symptoms stratified by participant background characteristics was determined using data from the questionnaire. HADS-A and D scores were validated as measures of anxiety and depression, respectively, with scores of 7 or less indicating negative, 8–10 indicating doubtful, and 11 or more indicating definite cases (14, 15). Associated factors were identified from a combination of univariate and multivariate logistic regression analyses, with PFH presence as the dependent variable. Univariate logistic regression analyses were performed to evaluate each participant’s background characteristics as an independent variable. Odds ratios (ORs), 95% confidence intervals (CIs), and *p*-values were calculated. A multivariate logistic regression analysis was performed using explanatory variables with *p*-values of <0.05 in the univariate analysis. Collinearity among explanatory variables was not addressed in this analysis, since the primary objective of the survey is to explore associated factors as broadly as possible in an exploratory manner and not to interpret relationships between the variables. A receiver operating characteristic (ROC) curve analysis (19) was conducted to estimate the optimal cutoff values of HADS scores for suspecting PFH. Statistical significance was determined using a two-sided alpha level of *p* of <0.05. All analyses were performed using R software (version 3.4.0 or higher), and data were tabulated using Microsoft Excel 2016.

## 3 Results

### 3.1 Participant composition and characteristics

**Figure 1:**
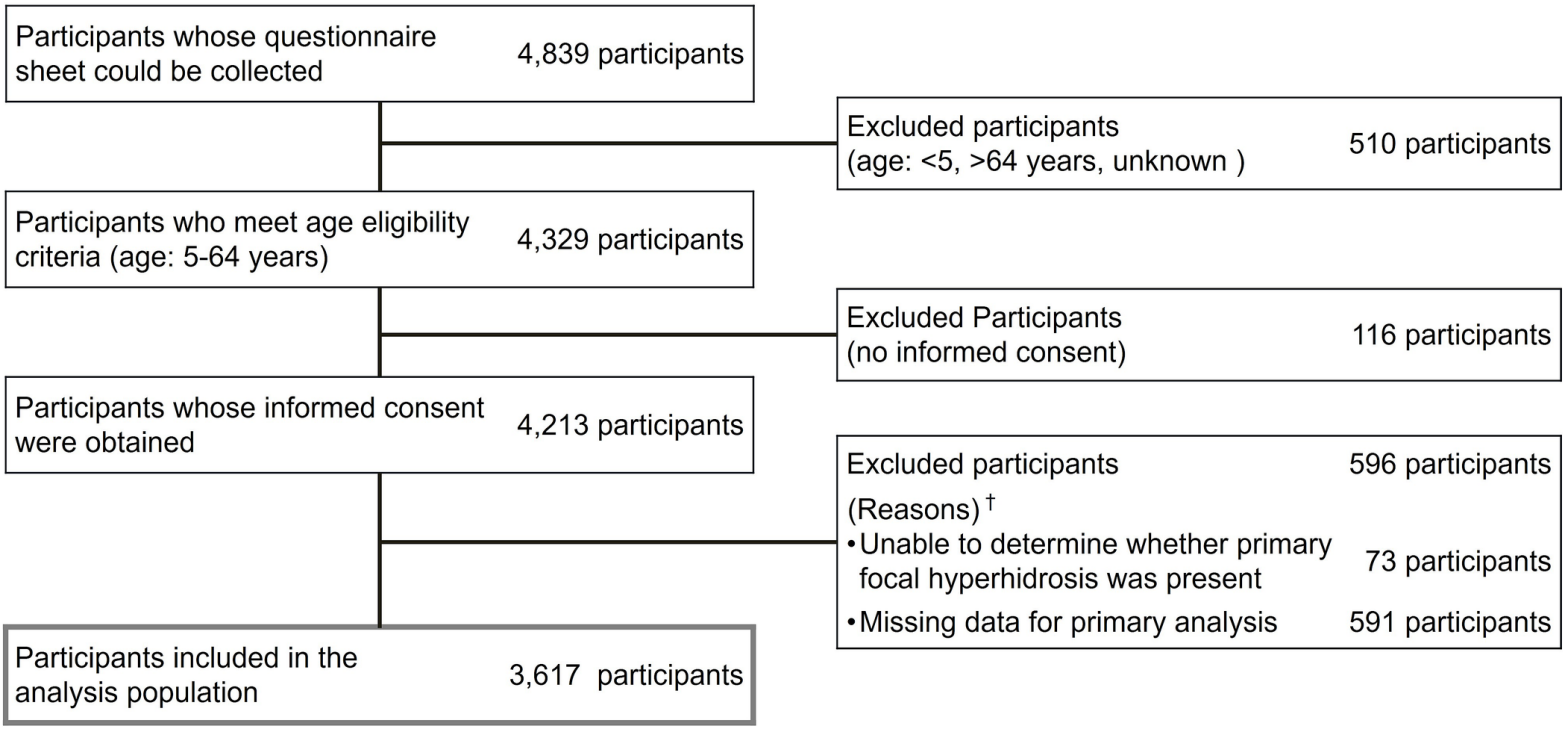
Participant composition. ^†^Contains duplicate.

A total of 3,617 participants were included in the analysis (Figure 1). The mean age ± SD was 38.5 ± 14.5 years, and 64.7% were female (Table 1).

**Table 1:** Participant characteristics.

Background factors	Statistics *n*/(%)	
Total	3,617	
Age; years		
Mean±SD	38.5 ± 14.5	
Median; IQR	40; 26–51	
<10	63	(1.7)
10–19	358	(9.9)
20–29	678	(18.7)
30–39	645	(17.8)
40–49	873	(24.1)
50–59	779	(21.5)
≥60	221	(6.1)
Sex		
Male	1,276	(35.3)
Female	2,341	(64.7)
HADS score		
HADS-A score (diagnosis of anxiety)		
Negative	2,459	(68.0)
Doubtful	661	(18.3)
Definite	497	(13.7)
HADS-D score (diagnosis of depression)		
Negative	2,551	(70.5)
Doubtful	665	(18.4)
Definite	401	(11.1)

Background factors	Absent *n*/(%)		Present *n*/(%)	
Lifestyle and daily habits				
Smoking^a^	3,049	(84.3)	568	(15.7)
Alcohol consumption^b^	1,837	(50.8)	1,780	(49.2)
Adequate sleep	1,147	(31.7)	2,470	(68.3)
Regular exercise^c^	2,307	(63.8)	1,310	(36.2)
Wet earwax	2,586	(71.5)	1,031	(28.5)
Skipping breakfast ≥3 days a week	2,788	(77.1)	829	(22.9)
Daily vegetable intake	877	(24.2)	2,740	(75.8)
Preference for Japanese-style meals	1,983	(54.8)	1,634	(45.2)
Daily use of VDTs				
<1 h			320	(8.8)
≥1–<2 h			545	(15.1)
≥2–<4 h			996	(27.5)
≥4–<6 h			693	(19.2)
≥6 h			1,063	(29.4)
Customer service occupation	1,850	(51.1)	1,767	(48.9)
Jobs requiring uniforms	2,131	(58.9)	1,486	(41.1)
Daily use of public transportation	2,325	(64.3)	1,292	(35.7)
Subjective symptoms				
Any of these symptoms	1.945	(53.8)	1.672	(46.2)
Headache	2,997	(82.9)	620	(17.1)
Dizziness	3,350	(92.6)	267	(7.4)
Low-grade fever	3,584	(99.1)	33	(0.9)
Palpitations	3,456	(95.5)	161	(4.5)
Decreased appetite	3,569	(98.7)	48	(1.3)
Constipation/diarrhea	3,087	(85.3)	530	(14.7)
Nausea	3,563	(98.5)	54	(1.5)
Tinnitus	3,363	(93.0)	254	(7.0)
Irritability/anxiety	3,173	(87.7)	444	(12.3)
Back and lower back pain	3,135	(86.7)	482	(13.3)
Neck and shoulder pain/stiffness	2,386	(66.0)	1,231	(34.0)
Numbness in the hands and feet	3,471	(96.0)	146	(4.0)
Irregular menstruation/erectile dysfunction	3,433	(94.9)	184	(5.1)
Shortness of breath	3,525	(97.5)	92	(2.5)
Dermatological conditions				
Any of these conditions	2,050	(56.7)	1,567	(43.4)
Atopic dermatitis	2,912	(80.5)	705	(19.5)
Acne	3,222	(89.1)	395	(10.9)
Axillary osmidrosis	3,605	(99.7)	12	(0.3)
Asteatosis (dry skin)	3,500	(96.8)	117	(3.2)
Psoriasis	3,541	(97.9)	76	(2.1)
Dandruff	3,572	(98.8)	45	(1.2)
Tinea infection (body, foot, and nail)	3,452	(95.4)	165	(4.6)
Rosacea	3,570	(98.7)	47	(1.3)
Seborrheic dermatitis	3,532	(97.6)	85	(2.4)
Skin cancer	3,615	(99.9)	2	(0.1)
Hand eczema	3,423	(94.6)	194	(5.4)
Non-dermatological conditions				
Any of these conditions	2,878	(79.6)	739	(20.4)
Cancer	3,581	(99.0)	36	(1.0)
Dyslipidemia	3,487	(96.4)	130	(3.6)
Migraine	3,530	(97.6)	87	(2.4)
Hypertension	3,377	(93.4)	240	(6.6)
Gastroesophageal reflux disease	3,562	(98.5)	55	(1.5)
Anxiety disorder	3,555	(98.3)	62	(1.7)
Depression	3,542	(97.9)	75	(2.1)
Type 2 diabetes mellitus	3,569	(98.7)	48	(1.3)
Bronchial asthma	3,498	(96.7)	119	(3.3)
Sleep apnea syndrome	3,592	(99.3)	25	(0.7)

a Present includes both current and former smokers.

b Present includes both daily drinkers and those who drink sometimes.

c Exercising 30 min/day, 2 times/week, for over a year.

IQR, interquartile range; HADS, Hospital Anxiety and Depression Scale; SD, standard deviation; VDT, visual display terminal.

### 3.2 Prevalence of PFH

**Figure 2:**
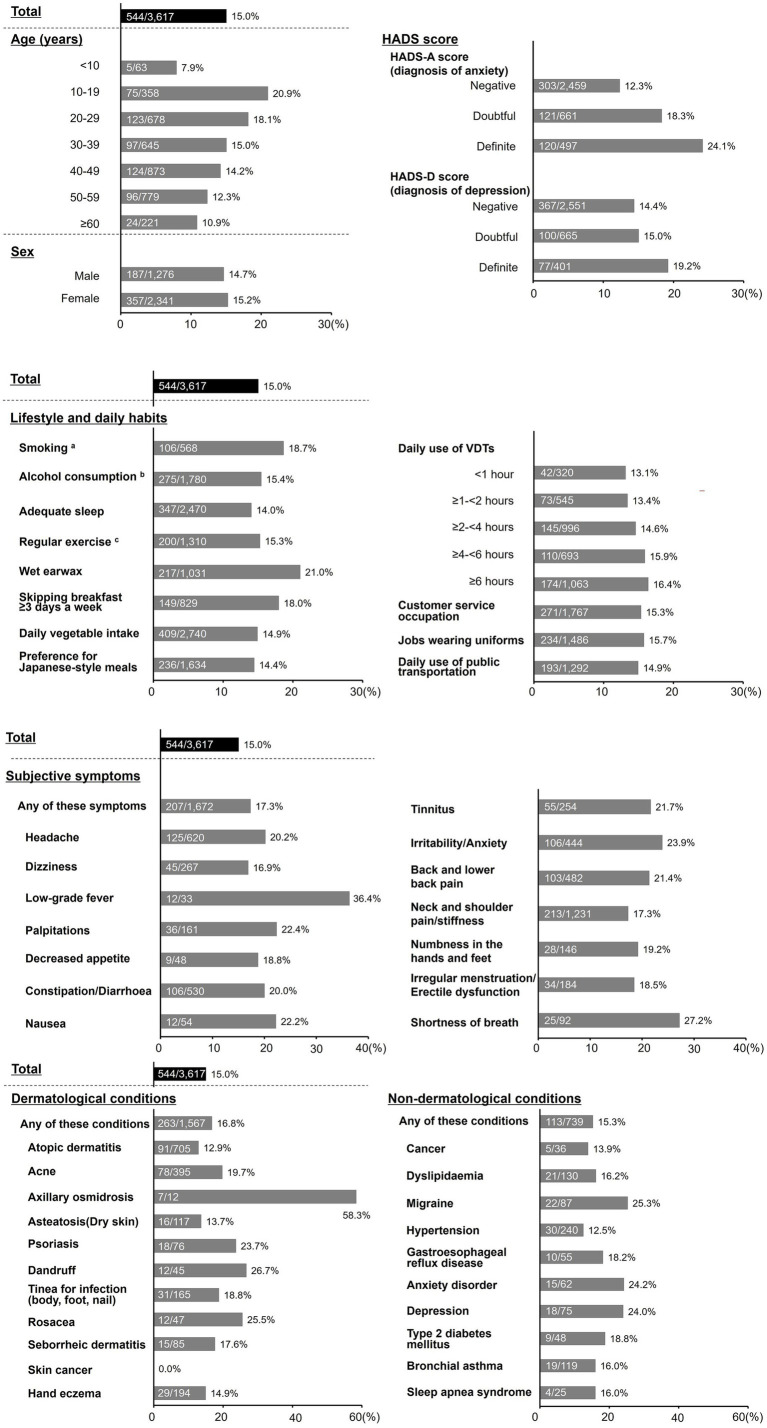
Prevalence of PFH. The numbers in the graph show the number of patients with PFH/subjects with each background factor and the prevalence of PFH (%). ^a^Present includes both current and former smokers. ^b^Present includes both daily drinkers and those who drink sometimes. ^c^Exercising 30 min/day, 2 times/week, for over a year. HADS, Hospital Anxiety and Depression Scale; PFH, primary focal hyperhidrosis; VDT, visual display terminal.

The overall prevalence of PFH in the analysis was 15.0%. Prevalence stratified by participants’ background characteristics is shown in Figure 2. A prevalence of >20% was observed in the participant subgroup aged 10–19 years and those with a definite HADS-A score, wet earwax, headache, low-grade fever, palpitations, constipation/diarrhea, nausea, tinnitus, irritability/anxiety, back and lower back pain, shortness of breath, axillary osmidrosis, psoriasis, dandruff, rosacea, migraine, anxiety disorder, and depression. We retrospectively chose this benchmark (>20%) to report 10–20 factors with a higher prevalence exceeding the overall prevalence of 15.0%.

### 3.3 Factors associated with PFH

A univariate logistic regression analysis revealed several potential factors associated with PFH, including age, HADS-A and HADS-D scores, and lifestyle and daily habits, such as smoking, inadequate sleep, wet earwax, and skipping breakfast ≥3 days a week; subjective symptoms, such as any symptoms, headache, low-grade fever, palpitations, constipation/diarrhea, tinnitus, irritability/anxiety, back and lower back pain, neck and shoulder pain/stiffness, and shortness of breath; dermatological conditions, such as any conditions, acne, axillary osmidrosis, psoriasis, dandruff, and rosacea; and non-dermatological conditions, such as migraine, anxiety disorder, and depression (Table 2). A multivariate logistic regression analysis was performed using explanatory variables identified as potential associated factors in the univariate analysis. Age, HADS-A score, smoking, wet earwax, back and lower back pain, axillary osmidrosis, and psoriasis were independently associated with PFH (Table 2).

**Table 2:** Univariate and multivariate analyses of factors associated with PFH.

Background factors	Univariate analysis		Multivariate analysis	
	OR (95%CI)	*p-*value	OR (95%CI)	*p-*value
Total				
Age (years)				
<10	0.325 (0.126–0.839)	0.020*	0.363 (0.137–0.962)	0.042*
10–19 (reference)	1.000		1.000	
20–29	0.836 (0.607–1.150)	0.275	0.684 (0.489–0.956)	0.026*
30–39	0.668 (0.478–0.933)	0.018*	0.507 (0.354–0.726)	<0.001*
40–49	0.625 (0.455–0.858)	0.004*	0.474 (0.332–0.676)	<0.001*
50–59	0.530 (0.380–0.739)	<0.001*	0.409 (0.283–0.593)	<0.001*
≥60	0.460 (0.280–0.754)	0.002*	0.407 (0.241–0.687)	0.001*
Sex				
Male (reference)	1.000		–	–
Female	1.050 (0.865–1.270)	0.633	–	–
HADS score				
HADS-A score (diagnosis of anxiety)				
Negative (reference)	1.000		1.000	
Doubtful	1.590 (1.270–2.010)	<0.001*	1.460 (1.140–1.870)	0.003*
Definite	2.260 (1.790–2.870)	<0.001*	1.780 (1.330–2.380)	<0.001*
HADS-D score (diagnosis of depression)		–	–	
Negative (reference)	1.000		1.000	
Doubtful	1.050 (0.829–1.340)	0.671	0.839 (0.648–1.090)	0.185
Definite	1.410 (1.080–1.860)	0.013*	0.872 (0.633–1.200)	0.405
Lifestyle and daily habits				
Smoking^a^	1.370 (1.080–1.730)	0.009*	1.450 (1.120–1.870)	0.005*
Alcohol consumption^b^	1.070 (0.888–1.280)	0.498	–	–
Adequate sleep	0.788 (0.651–0.954)	0.015*	0.940 (0.761–1.160)	0.563
Regular exercise^c^	1.030 (0.851–1.240)	0.773	–	–
Wet earwax	1.840 (1.520–2.230)	<0.001*	1.780 (1.470–2.160)	<0.001*
Skipping breakfast ≥3 days a week	1.330 (1.080–1.630)	0.007*	1.080 (0.861–1.350)	0.515
Daily vegetable intake	0.964 (0.781–1.190)	0.737	–	–
Preference for Japanese-style meals	0.918 (0.764–1.100)	0.362	–	–
Daily use of VDTs				
<1 h (reference)	1.000		–	–
≥1–<2 h	1.020 (0.681–1.540)	0.910	–	–
≥2–<4 h	1.130 (0.780–1.630)	0.523	–	–
≥4–<6 h	1.250 (0.851–1.830)	0.256	–	–
≥6 h	1.300 (0.901–1.860)	0.162	–	–
Customer service occupation	1.050 (0.872–1.260)	0.626	–	–
Jobs requiring uniforms	1.100 (0.913–1.320)	0.321	–	–
Daily use of public transportation	0.988 (0.816–1.190)	0.898	–	–
Subjective symptoms				
Any of these symptoms	1.480 (1.230–1.790)	<0.001*	1.050 (0.785–1.410)	0.737
Headache	1.550 (1.240–1.940)	<0.001*	1.160 (0.892–1.520)	0.264
Dizziness	1.160 (0.829–1.620)	0.389	–	–
Low-grade fever	3.280 (1.600–6.700)	0.001*	1.780 (0.835–3.800)	0.135
Palpitations	1.670 (1.140–2.450)	0.008*	1.060 (0.683–1.650)	0.787
Decreased appetite	1.310 (0.630–2.720)	0.470	–	–
Constipation/diarrhea	1.510 (1.190–1.910)	0.001*	1.150 (0.877–1.500)	0.315
Nausea	1.630 (0.851–3.110)	0.141	–	–
Tinnitus	1.620 (1.190–2.220)	0.002*	1.100 (0.778–1.570)	0.578
Irritability/anxiety	1.960 (1.540–2.490)	<0.001*	1.320 (0.989–1.760)	0.059
Back and lower back pain	1.660 (1.310–2.110)	<0.001*	1.420 (1.080–1.870)	0.012*
Neck and shoulder pain/stiffness	1.300 (1.080–1.570)	0.006*	1.070 (0.824–1.380)	0.620
Numbness in the hands and feet	1.360 (0.891–2.070)	0.155	–	–
Irregular menstruation/erectile dysfunction	1.300 (0.885–1.910)	0.182	–	-
Shortness of breath	2.160 (1.350–3.450)	0.001*	1.360 (0.805–2.310)	0.249
Dermatological conditions				
Any of these conditions	1.270 (1.060–1.520)	0.010*	0.982 (0.789–1.220)	0.870
Atopic dermatitis	0.805 (0.632–1.020)	0.078	-	-
Acne	1.460 (1.110–1.900)	0.006*	1.130 (0.819–1.560)	0.457
Axillary osmidrosis	8.000 (2.530–25.300)	<0.001*	5.440 (1.590–18.600)	0.007*
Asteatosis (Dry skin)	0.892 (0.522–1.520)	0.675	–	–
Psoriasis	1.780 (1.040–3.040)	0.036*	1.830 (1.030–3.260)	0.039*
Dandruff	2.080 (1.070–4.050)	0.032*	1.540 (0.744–3.170)	0.246
Tinea infection (body, foot, and nail)	1.330 (0.887–1.980)	0.169	–	–
Rosacea	1.960 (1.010–3.800)	0.047*	1.710 (0.848–3.460)	0.133
Seborrheic dermatitis	1.220 (0.691–2.140)	0.497	–	–
Skin cancer	0	0.962	–	–
Hand eczema	0.992 (0.662–1.490)	0.971	–	–
Non-dermatological conditions				
Any of these symptoms	1.020 (0.818–1.280)	0.831	–	–
Cancer	0.910 (0.352–2.350)	0.846	–	–
Dyslipidemia	1.090 (0.678–1.760)	0.718	–	–
Migraine	1.950 (1.190–3.190)	0.008*	1.170 (0.677–2.030)	0.571
Hypertension	0.796 (0.537–1.180)	0.256	–	–
Gastroesophageal reflux disease	1.260 (0.631–2.520)	0.512	–	–
Anxiety disorder	1.830 (1.010–3.290)	0.045*	0.938 (0.481–1.830)	0.851
Depression	1.810 (1.060–3.100)	0.031*	0.881 (0.477–1.630)	0.686
Type 2 diabetes mellitus	1.310 (0.630–2.720)	0.470	–	–
Bronchial asthma	1.080 (0.653–1.770)	0.774	–	–
Sleep apnea syndrome	1.080 (0.368–3.150)	0.893	–	–

Multivariate logistic regression analysis was performed using age (per 10 years), sex, and items with a *p*-value of less than 0.05 in the univariate analysis as adjustment factors. ^*^Statistically significant difference (*p* < 0.05).

a Present includes both current and former smokers.

b Present includes both daily drinkers and those who drink sometimes.

c Exercising 30 min/day, 2 times/week, for over a year.

CI; confidence intervals, HADS; Hospital Anxiety and Depression Scale, OR; odds ratio, PFH; primary focal hyperhidrosis, VDT; visual display terminal.

### 3.4 Cutoff value of HADS-A score for suspecting PFH

**Figure 3:**
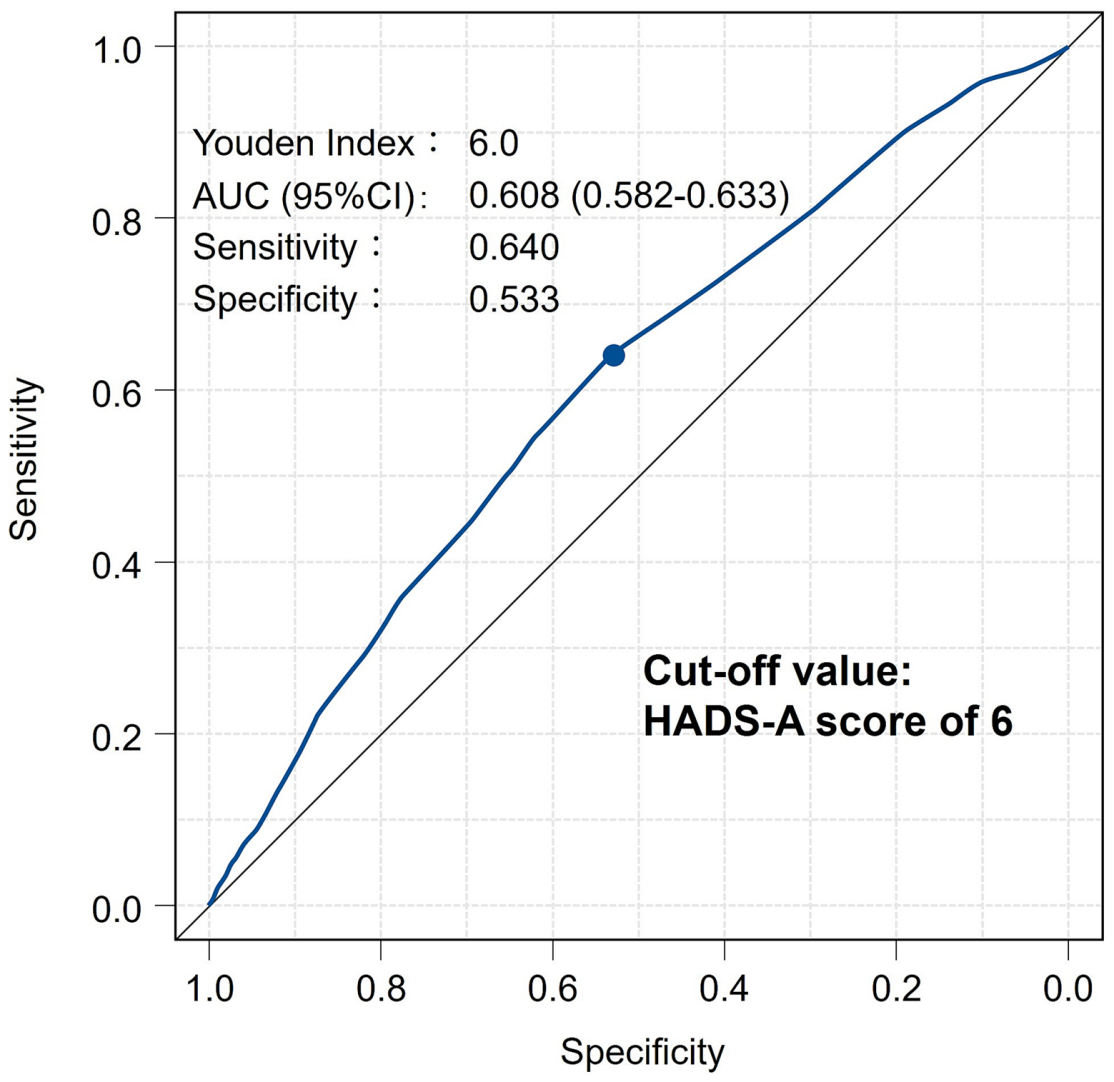
ROC curve showing optimal cutoff value of HADS-A score for suspecting PFH. AUC, Area under the curve; CI, confidence intervals; HADS, Hospital Anxiety and Depression Scale; PFH, primary focal hyperhidrosis; ROC, receiver operating characteristic.

The HADS-A score was identified as a significant associated factor for PFH in the multivariate analysis. Accordingly, an ROC curve analysis was conducted to estimate the optimal cutoff value of the HADS-A score for suspecting PFH. The cutoff value was determined to be 6 (Figure 3).

## 4 Discussion

In our questionnaire survey, the prevalence of PFH was 15.0% among 3,617 participants who visited 24 dermatological institutions in Japan in 2024. The prevalence was observed among a limited population of outpatients at the dermatological institutions rather than the general population, which therefore requires careful interpretation of the finding. A previous epidemiological web-based questionnaire survey conducted in Japan in 2020 reported a PFH prevalence of 10.0% among 60,969 individuals registered in online survey panels (2). Unlike the previous study, our survey population consisted of patients who sought care at dermatology clinics and therefore may have included a higher proportion of individuals with PFH or those at greater dermatological risk for PFH. The findings of our survey suggest that the majority of patients visiting dermatological institutions in Japan experience PFH symptoms in real-world clinical practice.

Our survey determined the prevalence of PFH symptoms stratified by various types of participant background characteristics. Physicians may consider the possibility of PFH in patients who exhibit background characteristics with a higher prevalence (e.g., >20%), as shown in the Results section. In our survey, the highest prevalence was observed among teenagers, with a decreasing trend as age increased. This age-related pattern differs from the finding of a previous epidemiological survey conducted during the period of 2009–2010 among individuals of 20 companies and schools, which reported that PFH prevalence peaked at 20 years and declined thereafter (18). Considering the fact that the prevalence of participants aged more than 20 years aligns with trends reported in the previous survey, the higher prevalence among teenagers in our study may reflect an increased burden of PFH among adolescents who seek care at dermatological institutions compared to the general population. A previous cross-sectional web-based survey of 1,080 university students in Japan (mean age: 18.8 years) reported that the prevalence of hyperhidrosis that satisfied its diagnostic criteria (13) was 11%. In that study, compared to participants who screened negative for hyperhidrosis, the OR of anxiety induced by sweating was 7.11 (95% CI: 3.99–12.65) for mild/moderate hyperhidrosis and 23.46 (95% CI: 7.15–76.93) for severe hyperhidrosis, (20). These findings indicate that many teenage individuals suffer from PFH symptoms and have a high risk of anxiety, emphasizing the need for increased disease awareness among younger individuals.

A combination of univariate and multivariate logistic regression analyses exploratively identified several factors associated with PFH. Among them, the ORs were higher in order of axillary osmidrosis (OR = 5.440), psoriasis (OR = 1.830), wet earwax (OR = 1.780), definite HADS-A score (OR = 1.780), doubtful HADS-A score (OR = 1.460), smoking (OR = 1.450), and back and lower back pain (OR = 1.420). When individuals or patients who visit the medical institutions present with these background characteristics, screening for PFH may help identify potential undiagnosed patients with PFH, even if the patients visit for the management of diseases other than PFH. A previous retrospective chart review of 723 Japanese patients with axillary osmidrosis has shown that almost all patients (96.1%) have wet earwax, with a frequency much higher than that in the general Japanese population. Additionally, hyperhidrosis is observed in 61.8% of these patients (21). These findings support the validity of axillary osmidrosis and wet earwax as factors associated with PFH, which are consistent with our survey results. Stress is a well-known risk factor for a common chronic inflammatory skin disease such as psoriasis (22), and elevated social stress levels have also been reported among patients with PFH (23). Stress stimulates the cerebral cortex and limbic system, activating the psychogenic sweating center in the hypothalamus (24), and visible skin disease, such as PFH, causes psychological stress in patients (25), affecting each other in a bidirectional manner. Therefore, it is plausible that patients with PFH are more likely to have comorbid psoriasis, potentially due to shared stress-related mechanisms, which may explain why psoriasis was identified as a significant associated factor in our analysis. HADS-A score is a validated self-assessment scale for detecting anxiety in outpatient settings (14, 15). Several reports have indicated an association between hyperhidrosis and anxiety (10, 20, 26–29). Interestingly, only definite and doubtful HADS-A scores were identified as significant associated factors for PFH in our analysis, whereas a diagnosis of anxiety disorder was not. This finding suggests that the HADS-A score may be more directly associated with PFH than with the formal diagnosis of an anxiety disorder. The questions for the HADS-A score include “Do you ever feel tense?” “Do you worry a lot?” “Do you have panic attacks?” and “Do you feel something awful is about to happen?” (14). Patients with PFH may experience emotions detectable through these question items. In contrast, a previous systematic review including 32 studies with 12,812 participants has reported that the prevalence of anxiety in dermatology outpatient clinics is 26.7% (95% CI: 22.4–31.4%) (30). Our multivariate analysis did not identify anxiety disorder as an independent associated factor for PFH. This may be attributable to the inclusion of many dermatology outpatients with various comorbid conditions in our study population and the statistical adjustment for dermatological conditions such as acne, axillary osmidrosis, psoriasis, dandruff, and rosacea. Smoking is known to induce sweating via nicotinic receptor activation, which results in the augmentation of muscarinic receptor-mediated eccrine sweating (31). Several human studies have also reported an association between smoking and hyperhidrosis (32, 33). However, the relationship between PFH and back and lower back pain remains unclear.

Our survey detected a HADS-A score as one of the factors associated with PFH, and anxiety is a well-known risk factor for the disease (10, 20, 26–29). As mentioned in the Introduction section, PFH is one of the visible skin diseases that imposes a significant impact on patients’ social lives (visible stigma) (5). Visible skin diseases trigger a vicious cycle involving stigma recognition, social evaluation, anxiety/shame, safety behaviors/avoidance, and reinforcement of anxiety, leading to a significant decline in patients’ QOL (5, 34). Several recent and relevant studies have reported that visible dermatological conditions—such as vitiligo, seborrheic dermatitis, alopecia areata, acne, verruca vulgaris, and contact dermatitis—are strongly associated with social appearance anxiety, even in the absence of formal psychiatric diagnoses (35–40). A management approach of psychodermatology is required for patients with PFH, considering the burden patients face from such a significant vicious cycle. For instance, being open to the experiences of stigma (as opposed to avoidance), maintaining distance from stigmatized thoughts (as opposed to self-stigmatizing), and bringing attention to value-based committed actions (as opposed to passivity) are reported to contribute to less stigmatized experiences (41).

The optimal cutoff value of the HADS-A score for suspecting PFH was estimated to be 6 based on the ROC curve analysis. The score was validated as a measure of anxiety, with scores of 7 or less indicating negative, 8–10 indicating doubtful, and 11 or more indicating definite cases (14, 15). Interestingly, a score of 6, which indicates the negative range for anxiety disorder, was identified as the optimal cutoff value for suspecting PFH. This finding warrants further investigation, as there is no existing theoretical evidence to support it. We speculate that subclinical anxiety in PFH, as detected by the HADS-A score, may be related to chronic visibility, anticipatory social stress, and appearance-related self-consciousness, all of which can be triggered by visible skin disease. However, there are no data confirming that the HADS-A specifically captures appearance-related anxiety in visible skin disease. Thus, although this finding has limitations in interpretation, as mentioned above, the HADS-A consists of seven simple items (14), and it may still serve as a useful tool for detecting unmedicated patients with PFH in dermatology outpatient care. We also believe that a tool for assessing social appearance anxiety, such as the Social Appearance Anxiety Scale (42), may be more useful for detecting visible skin disease, such as PFH; however, it was not possible to investigate this in the present survey. Based on our findings, specific clinical strategies may be proposed, including screening PFH patients for psychosocial distress, integrating brief anxiety- or appearance-related screening tools, and considering interdisciplinary management involving both dermatology and mental health.

A key strength of this questionnaire-based survey is its large sample size, which included 3,617 participants. However, a limitation is that, because participants were patients who visited dermatological institutions, the findings may not be generalizable to the broader population, particularly to individuals without dermatological conditions. The 24 dermatological institutions that participated in this study were located in a limited area near Kobe City, Japan. Therefore, the possibility of selection bias in the study institutions cannot be ruled out. Additionally, the study was conducted over a 4-month period, from April to July 2024, and potential seasonal variations in hyperhidrosis occurrence were not considered. Patients’ background factors, including disease conditions, were assessed based on self-reported information and used to explore associated factors. Therefore, the findings should be interpreted with caution. In this study, the HADS, which measures depression and anxiety, was applied across a wide age range (5–64 years). However, the Japanese version of the HADS is a scale whose reliability and validity have been primarily verified in adults aged 18 years and older (43). Therefore, analyses that include data from participants under 18 years old may not accurately reflect the influence of the HADS as a factor associated with PFH. Finally, collinearity among explanatory variables was not addressed in our study, and our exploratory findings require validation through analyses that account for multicollinearity. Thus, the associated factors in this study may help detect unmedicated potential patients with PFH during routine clinical practice, although further verification is required to establish whether these factors represent causal risk factors or reflect the pathophysiological state of the disease.

## 5 Conclusion

The prevalence of PFH was 15.0% among participants who visited dermatological institutions in Japan in 2024, based on a multi-center, cross-sectional questionnaire survey of 3,617 participants. Axillary osmidrosis, psoriasis, wet earwax, HADS-A score, smoking, back and lower back pain, and age were exploratively identified as factors associated with PFH in this outpatient population. The optimal cutoff value of the HADS-A score for suspecting PFH was estimated to be 6, suggesting a potential association between subthreshold anxiety and PFH. These findings may help in identifying unmedicated patients during routine medical practice and support active interventions, thereby improving the quality of life and well-being of individuals with PFH.

## Data Availability

The raw data supporting the conclusions of this article will be made available by the authors, without undue reservation.
